# How Is Wildlife Affected by the COVID-19 Pandemic? Lockdown Effect on the Road Mortality of Hedgehogs

**DOI:** 10.3390/ani11030868

**Published:** 2021-03-18

**Authors:** Rafał Łopucki, Ignacy Kitowski, Magdalena Perlińska-Teresiak, Daniel Klich

**Affiliations:** 1 Centre for Interdisciplinary Research, The John Paul II Catholic University of Lublin, Konstantynów 1J, 20-708 Lublin, Poland; lopucki@kul.lublin.pl; 2 State School of Higher Education in Chełm, Pocztowa 54, 22-100 Chełm, Poland; ignacyk@autograf.pl; 3 Department of Animal Genetics and Conservation, Warsaw University of Life Sciences-SGGW, Ciszewskiego 8, 02-786 Warsaw, Poland; magdalena_perlinska_teresiak@sggw.edu.pl

**Keywords:** coronavirus, anthropause, road traffic, roadkill, population, Erinaceus

## Abstract

**Simple Summary:**

In 2020, many countries around the world went into lockdown to limit the spread of COVID-19, and many people substantially reduced their outdoor activity and began socially distancing to avoid infection. Such rapid and widespread changes in the lives of people all over the world must have an impact on the environment and wildlife. Most of the current pandemic–wildlife papers focus on changes in the movement and behavior of wild animals. These changes, although interesting and worth documenting, may be ephemeral and may not have any significant effect on wildlife in the long run; they may only show how our daily presence may limit the presence and behavior of animals. In this paper, we suggest that scientific interest should be directed toward more permanent changes in the functioning of wildlife. One such topic is the lower road mortality rates of animals during the lockdown. In this study, hedgehog roadkill levels during the lockdown were over 50% lower than in the pre-pandemic years. Reduced road mortality in the case of hedgehogs may mean tens of thousands of survivors nationwide. We hypothesize that this may result in a change in the demographic and genetic characteristics of the population of hedgehogs, and also help to stop the long-term decline in the number of hedgehogs in Europe.

**Abstract:**

Globally, wildlife is affected by unprecedented changes related to the COVID-19 pandemic. In this paper, the lockdown effect on the traffic-related mortality in hedgehogs in an urban area was studied. Comparing the pre-pandemic (2018 and 2019) and pandemic (2020) years, we showed that hedgehog roadkill levels during the lockdown period were over 50% lower (which means a decrease greater than the decrease in road traffic in the same period measured by the number of accidents or the average number of vehicles per day). Based on literature data, we showed that this may mean at least tens of thousands of hedgehogs have survived on a national scale. We report the need to start intensive research on the possible demographic and genetic effects of this unique phenomenon. We also ask how stable the effect of the COVID-19 pandemic will be on wildlife and whether the lockdown (which is an anthropause) may reverse the negative trends in the decline in the number of wild species, including hedgehogs.

## 1. Introduction

In 2020, many countries around the world went into lockdown to limit the spread of COVID-19. The tragic news about the growing number of infections and deaths coming from different parts of the globe caused many people to substantially reduce their outdoor activity and begin socially distancing to avoid infection. Other activities were also promoted by many governments: the remote work and education model was introduced on a large scale, some sectors of the economy were closed, and staying at home was recommended.

Such rapid and widespread changes in the lives of people all over the world must have an impact on the environment and wildlife [1]. Changes in air quality related to the concentration of traffic-related black carbon, carbon monoxide, nitrogen dioxide, and particulate matter PM_2.5_ and PM_10_ quickly began to be reported from many countries around the world [2,3,4,5]. Environmental noise reduction [6,7] and improvement to the ecological condition of highly affected beaches due to the lack of tourists were also reported [8].

Interestingly, the response of wildlife to changes in the functioning of human society and economy was also rapid. The first phenomenon noticed was that the reduction in human disturbance allowed wildlife to exploit built-up habitats and to increase daily activity [9,10,11]. Since the beginning of the pandemic, evidence of the presence of wild animal species in areas where they have not been seen for a long time has been shared on social media [7,12,13]. A number of various changes in animal behavior have also been observed, e.g., in birds’ vocalizations during the COVID-19 quietus [7], increased aggression, changes in feeding sites, and the formation of new competitive systems in synanthropic species suddenly deprived of anthropogenic food [10,14]. During the pandemic, people have been given the opportunity to gain unanticipated insight into how their presence affects animal behavior and how quickly and flexibly animals can react to unprecedented changes, such as lockdown and the “global human confinement experiment” [1,15].

However, a question arises as to how persistent the current changes in nature will be and what further ecological consequences they will have on the populations of wild species [16]. Due to the short duration of this specific anthropause (the World Health Organization declared the pandemic on 11 March 2020), the data showing the impact of the lockdown on key population parameters, such as abundance, mortality, reproduction, or gene flow, are very sparse [9]. More research is required to document changes that may affect the vital characteristics of a population, particularly in species with alarming declines in numbers. We hope that for such (often rare and protected) species, the lockdown period may unexpectedly become a breakthrough moment in reversing negative population trends.

In this paper, we explored whether lockdown may significantly reduce road wildlife mortality due to the reduced outdoor activity of people and the reduced car traffic. We used the hedgehog as a model species, as it is mentioned in many papers as one of the most frequent victims of car traffic, especially in urban areas [17,18]. Given the decreasing abundance of hedgehogs in Europe [19,20,21,22,23], quantification of the decrease in roadkill during the COVID-19 pandemic is necessary for proper understanding its population trends, proper planning of research, and conservation activities.

## 2. Materials and Methods

### 2.1. Study Sites and Estimation of Hedgehog Roadkills

The study was carried out in the city of Chełm (51°07′ N, 23°28′ E), Poland, Europe. The city has an area of 35.5 km^2^, a population of 65,643, 160 km of paved roads, and over 55,000 registered vehicles (in 2018–2020) [24]. Small cities are much more common than large cities (in number and total occupied area), and almost half of the world’s urban population lives in small cities [25,26].

In Chełm, the monitoring of roadkill of the northern white-breasted hedgehog (*Erinaceus roumanicus*) has been conducted for several years in seven permanent study sites (selected on the basis of previous long-term observations). In 2020, the monitoring was continued despite the pandemic.

**Figure 1:**
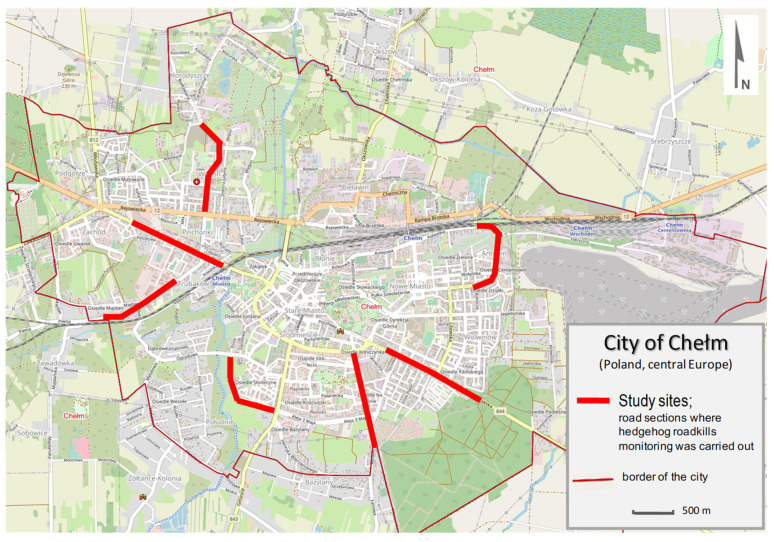
Location of study sites in the city of Chełm, where the monitoring of hedgehog roadkill was conducted.

Each study site includes asphalt roads about 1.2 km in length (from 1.07 to 1.34 km) located in different parts of the city (Figure 1). The sites were monitored once a week from March to July by the same walking observers [27]. The observers were two scientists familiar with the regime of ecological research. The inspections were performed in the morning between 6:00 a.m. and 9:00 a.m. The inspection of a single study site lasted approximately 20 min. The number of dead animals found on an asphalt road and in its vicinity (up to 1.5 m) was recorded, and dead animals were removed to avoid double counts.

**Figure 2:**
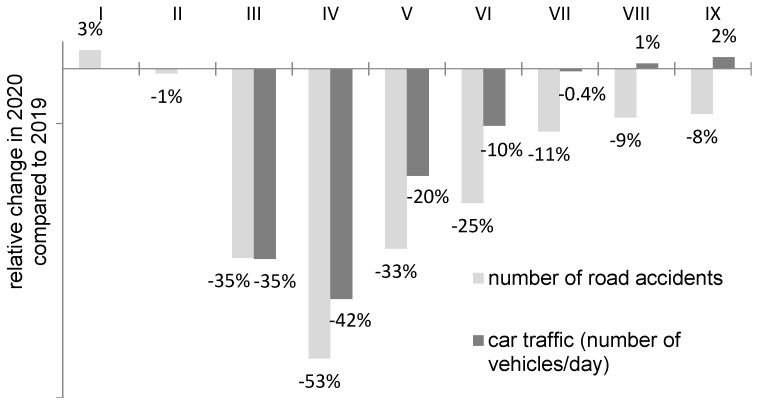
Relative change in road traffic (March–September) and the number of road accidents (January–September) in 2020 compared to 2019 in Poland. Since March, there has been a sharp decline in road traffic (and road accidents) due to the lockdown.

For the purposes of this study, data from three consecutive years, namely, 2018, 2019, and 2020, were used. The data from 2018 and 2019 are reference values and show the typical number of dead hedgehogs recorded in the monitored sites (mean dead hedgehogs/site/year = 5.14; SD = 1.61; range = 3–9). The data obtained for 2020 cover the reduced car traffic period during the first lockdown in Poland (Figure 2).

### 2.2. Impact of the Lockdown on Car Traffic

**Figure 3:**
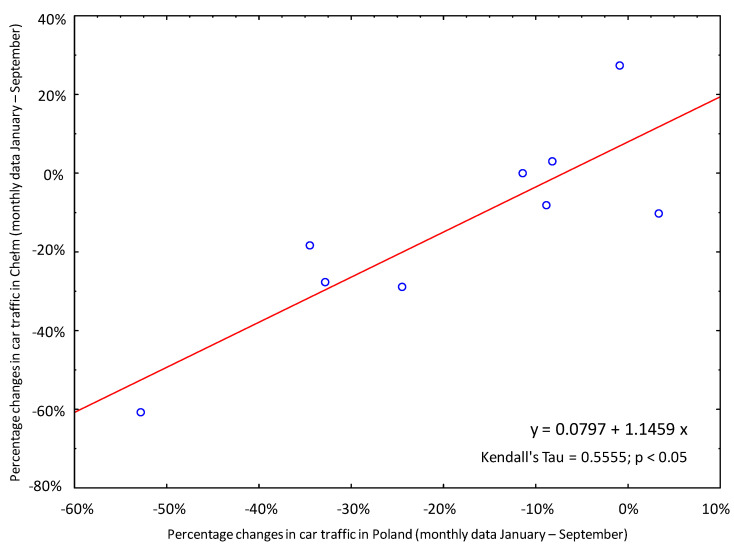
Local data of the reduction in car traffic from Chełm correlated with the national data. Monthly data (January–September) from Chełm (Y-axis) showing the percentage change in the number of road accidents and collisions in 2020 compared to 2019, and nationwide data (X-axis) showing the percentage change in the number of road accidents in 2020 compared to 2019 are presented.

During the lockdown in Poland, a significant reduction in car traffic was observed, as in other countries [3,28,29,30,31,32]. The following data were used as a measure of this change:

1. Nationwide data from automatic traffic measurement stations (measuring the number of vehicles per day) showing the percentage change in car traffic in 2020 compared to 2019, from March to September [33] (Figure 2);
2. Nationwide data showing the percentage change in the number of road accidents in 2020 compared to 2019, from January to September [34] (Figure 2);
3. Local data from Chełm showing the percentage change in the number of road accidents and collisions in 2020 compared to 2019, from January to September (source: the Polish police in Chełm). The local data correlated with the national data (Kendall’s tau rank correlation coefficient = 0.55; *p* < 0.05) and are presented in a separate figure (Figure 3).

### 2.3. Statistical Analysis

Nonparametric one-way ANOVA with ranks (Kruskal–Wallis test) was used to compare the number of hedgehog roadkills per year in the seven study sites in 2018, 2019, and 2020. For the purposes of the analysis, we summed up all dead hedgehogs found in a given study site in a given year and thus obtained 21 data points (seven for each year). Pairwise comparisons between individual years were made using a post-hoc test (z-value) using mean ranks. Analyses were performed using Statistica v13 software.

## 3. Results

**Figure 4:**
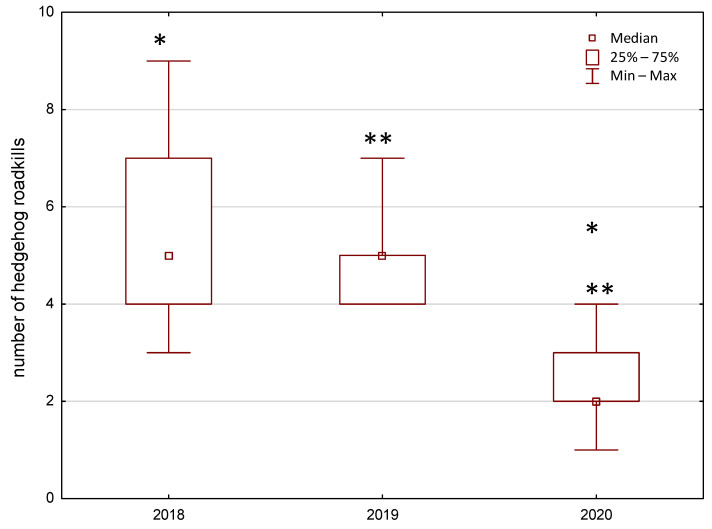
Number of hedgehog roadkills (in March–July) in seven study sites in the city of Chełm, (Poland, Europe) during the pre-pandemic period (2018 and 2019) and during the lockdown period (2020). Significant differences: * *p* ≤ 0.01 and ** *p* = 0.01.

In 2018 and 2019, the same median values of hedgehog roadkills in each of the study sites were recorded, i.e., five carcasses (Figure 4). On average, there were 5.43 (SD = 2.07) and 4.86 (SD = 1.07) hedgehog roadkills in each of the study sites in 2018 and 2019, respectively. In 2020, however, the median number of roadkills decreased to two carcasses per study site (the average number carcasses/site/year = 2.42; SD = 0.97). The Kruskal–Wallis ANOVA test showed that the “year” factor significantly influenced the observed number of hedgehog roadkills (H[2] = 11.45; *p* < 0.01; N = 21). The post-hoc tests showed that the average number of roadkills in 2018 and 2019 did not differ significantly; however, significant differences were noted between 2018 and 2020 (z = 2.95; *p* < 0.01) and between 2019 and 2020 (z = 2.80; *p* = 0.01) (Figure 4).

## 4. Discussion

Despite the numerous media reports and the growing number of scientific papers documenting the impact of the global lockdown on the unusual behavior of animals [1,7,10,12,13,15], studies documenting changes in fertility, mortality, or genetic effects are still scarce [9]. This is understandable, as it is usually difficult to observe these effects on such population parameters in a short time scale.

In the case of the analyzed species, however, this effect can be reliably shown, as traffic-related mortality of hedgehogs is a well-known and widely described phenomenon [35,36,37,38,39,40]. Hedgehogs die on roads throughout the entire year, except during winter hibernation, and the peak mortality is during the summer months. Males are more often the victims, one reason being the promiscuous mating system of hedgehogs [38,41]. The number of hedgehog roadkills in absolute numbers on a national scale is high: 167,000–335,000 hedgehog road casualties annually in Great Britain [42], 113,000–340,000 in the Netherlands [43], and 230,000–350,000 in Belgium [44]. However, it is not clear what proportion of the annual loss of the hedgehog population may be caused by road mortality. Various authors have estimated that traffic collisions may cause a loss of 3%–24% of a local hedgehog population and 9%–30% of a nationwide population [18].

Could the decrease in road deaths during the pandemic be important for the hedgehog population? It seems so, since roadkill is consistently in the top three most commonly recorded causes of death in hedgehogs (alongside illness and natural predation), which is consistent with the hypothesis that traffic mortality potentially exerts substantial pressure on population dynamics [18,40]. This pressure can be both quick and direct (decline in numbers by death of individual animals), as well as indirect with effects shifted in time (by effecting reproduction, migration rates, and genetic diversity) [18,45,46,47,48,49]. In this study, the mortality rate during the studied period (March–July) was reduced by over 50%. This means that the number of roadkills decreased more than road traffic did, as measured by the number of accidents or the average number of vehicles per day in the same period (Figure 2). Taking into account the above-cited estimates reported by Huijser and Bergers [43], Holsbeek et al. [44], and Wembridge et al. [42], this means that at least tens of thousands of hedgehogs may survive in the country as a result of the lockdown. The demographic, genetic, and conservation consequences of this unexpected and unusual phenomenon may be diverse. However, this phenomenon, like the reactions of other animal species, requires further appropriately targeted research [16]. Furthermore, it should be borne in mind that our results are based on data from one city and its seven study sites, and for other cities, the results for the decline in the road mortality of hedgehogs during the lockdown may be different. In future studies on the population effects of increased hedgehog survival in 2020, the appropriate method is of key importance to ensure correct inference, since the mortality of hedgehogs on roads is often inferred from reports of finding a dead hedgehog by volunteers. Such data can be the basis for illustrating population trends only on the assumption that the social interest in the project is relatively constant. Increasing or decreasing popularity of the social initiative may be reflected in an upward or downward trend in reported cases. In the event of the COVID-19 pandemic, such an unusual impact may unfortunately have occurred. Many social observers may have decreased their outdoor activity and may have been less likely to encounter the roadkill of wildlife. There may also have been less interest in the voluntary collection of ecological data in the wake of the tragic reports of human mortality rates. Therefore, the lower number of reports does not necessarily mean a lower mortality rate of hedgehogs. Administrators of national wildlife roadkill monitoring projects reported by members of the public (e.g., projectsplatter.co.uk) emphasize the problem with the interpretation of civic data from the pandemic period.

Comparative data from permanent study sites monitored by professional researchers (as in this study) are needed to correctly estimate the short- and long-term lockdown effect on hedgehog population parameters. The widest possible group of hedgehog researchers with comparative pre-pandemic data should include such research tasks in their plans. It is also crucial to take into account the recommendations regarding the influence of carcass persistence and detectability on the obtained results and recommendations for car monitoring [27,50]. We suggest that, although they are the most time-consuming approaches, walking surveys in permanent study sites are the best method of studying the roadkill of small-sized animals.

## Data Availability

The data presented in this study are available on request from the corresponding author.

## References

[1] Bates A.E., Primack R.B., Moraga P., Duarte C.M. (2020). COVID-19 pandemic and associated lockdown as a “Global Human Confinement Experiment” to investigate biodiversity conservation. Biol. Conserv..

[2] Bao R., Zhang A. (2020). Does lockdown reduce air pollution? Evidence from 44 cities in northern China. Sci. Total Environ..

[3] Hudda N., Simon M.C., Patton A.P., Durant J.L. (2020). Reductions in traffic-related black carbon and ultrafine particle number concentrations in an urban neighborhood during the COVID-19 pandemic. Sci. Total Environ..

[4] Nakada L.Y.K., Urban R.C. (2020). COVID-19 pandemic: Impacts on the air quality during the partial lockdown in São Paulo state, Brazil. Sci. Total Environ..

[5] Sharifi A., Khavarian-Garmsir A.R. (2020). The COVID-19 pandemic: Impacts on cities and major lessons for urban planning, design, and management. Sci. Total Environ..

[6] Basu B., Murphy E., Molter A., Sarkar Basu A.S., Sannigrahi S. (2020). Investigating changes in noise pollution due to the COVID-19 lockdown: The case of Dublin, Ireland. Sustain. Cities Soc..

[7] Derryberry E.P., Phillips J.N., Derryberry G.E., Blum M.J., Luther D. (2020). Singing in a silent spring: Birds respond to a half-century soundscape reversion during the COVID-19 shutdown. Science.

[8] Zambrano-Monserrate M.A., Ruano M.A., Sanchez-Alcalde L. (2020). Indirect effects of COVID-19 on the environment. Sci. Total Environ..

[9] Manenti R., Mori E., Di Canio V., Mercurio S., Picone M. (2020). The good, the bad and the ugly of COVID-19 lockdown effects on wildlife conservation: Insights from the first European locked down country. Biol. Conserv..

[10] Rutz C., Loretto M.C., Bates A.E., Davidson S.C., Duarte C.M. (2020). COVID-19 lockdown allows researchers to quantify the effects of human activity on wildlife. Nature Ecol. Evol..

[11] Silva-Rodríguez E.A., Gálvez N., Swan G.J.F., Cusack J.J., Moreira-Arce D. (2020). Urban wildlife in times of COVID-19: What can we infer from novel carnivore records in urban areas?. Sci. Total Environ..

[12] Abd Rabou A.N. (2020). How Is the COVID-19 Outbreak Affecting Wildlife around the World?. Open J. Ecol..

[13] Bar H. (2020). COVID‑19 lockdown: Animal life, ecosystem and atmospheric environment. Environ. Dev. Sustain..

[14] Gilby B.L., Henderson C.J., Olds A.D., Ballantyne J.A., Bingham E.L. (2021). Potentially negative ecological consequences of animal redistribution on beaches during COVID-19 lockdown. Biol. Conserv..

[15] Montgomery R.A., Raupp J., Parkhurst M. (2021). Animal Behavioral Responses to the COVID-19 Quietus. Trends Ecol. Evol..

[16] Zellmer A.J., Wood E.M., Surasinghe T., Putman B.J., Pauly G.B. (2020). What can we learn from wildlife sightings during the COVID-19 global shutdown?. Ecosphere.

[17] Grilo C., Sousa J., Ascensão F., Matos H., Leitão I. (2021). Individual spatial responses towards roads: Implications for mortality risk. PLoS ONE.

[18] Moore L.J., Petrovan S.O., Baker P.J., Bates A.J., Hicks H.L. (2020). Impacts and Potential Mitigation of Road Mortality for Hedgehogs in Europe. Animals.

[19] Krange M. (2015). Change in the Occurrence of the West European Hedgehog (*Erinaceus europaeus*) in Western Sweden during 1950–2010. Master’s Thesis.

[20] Van de Poel J.L., Dekker J., Van Langevelde F. (2015). Dutch hedgehogs *Erinaceus europaeus* are nowadays mainly found in urban areas, possibly due to the negative effects of badgers *Meles meles*. Wildl. Biol..

[21] Hof A.R., Bright P.W. (2016). Quantifying the long-term decline of the West European hedgehog in England by subsampling citizen-science datasets. Eur. J. Wildl. Res..

[22] Pettett C.E., Johnson P.J., Moorhouse T.P., Macdonald D.W. (2012). National predictors of hedgehog *Erinaceus europaeus* distribution and decline in Britain. Mamm. Rev..

[23] Taucher A.L., Gloor S., Dietrich A., Geiger M., Hegglin D., Bontadina F. (2020). Decline in Distribution and Abundance: Urban Hedgehogs under Pressure. Animals.

[24] Łopucki R., Kitowski I. (2017). How small cities affect the biodiversity of ground-dwelling mammals and the relevance of this knowledge in planning urban land expansion in terms of urban wildlife. Urban. Ecosyst..

[25] Kendal D., Egerer M., Byrne J.A., Jones P.J., Marsh P. (2020). City-size bias in knowledge on the effects of urban nature on people and biodiversity. Environ. Res. Lett..

[26] Łopucki R., Klich D., Kitowski I., Kiersztyn A. (2020). Urban size effect on biodiversity: The need for a conceptual framework for the implementation of urban policy for small cities. Cities.

[27] Santos R.A.L., Santos S.M., Santos-Reis M., Picanço de Figueiredo A., Bager A. (2016). Carcass persistence and detectability: Reducing the uncertainty surrounding wildlife-vehicle collision surveys. PLoS ONE.

[28] Aloi A., Alonso B., Benavente J., Cordera R., Echániz E. (2020). Effects of the COVID-19 Lockdown on Urban Mobility: Empirical Evidence from the City of Santander (Spain). Sustainability.

[29] Barnes S.R., Beland L.P., Huh J., Kim D. (2020). The Effect of COVID-19 Lockdown on Mobility and Traffic Accidents: Evidence from Louisiana.

[30] Katrakazas C., Michelaraki E., Sekadakis M., Yannis G. (2020). A descriptive analysis of the effect of the COVID-19 pandemic on driving behavior and road safety. TRIP.

[31] Qureshi A.I., Huang W., Khan S., Lobanova I., Siddiq F. (2020). Mandated societal lockdown and road traffic accidents. Accid Anal. Prev..

[32] Saladié O., Bustamante E., Gutiérrez A. (2020). COVID-19 lockdown and reduction of traffic accidents in Tarragona province, Spain. TRIP.

[33] General Director for National Roads and Motorways. www.gddkia.gov.pl.

[34] The Polish Police and Polish Road Safety Observatory Website. www.observatoriumbrd.pl.

[35] Kristiansson H. (1990). Population variables and causes of mortality in a hedgehog (*Erinaceus europaeus*) population in southern Sweden. J. Zool.

[36] Huijser M.P., Bergers P.J.M. (1998). Platte egels tellen: Resultaten van een VZZ-actie. Zoogdier.

[37] Rondinini A., Doncaster C.P. (2002). Roads as barriers to movement for hedgehogs. Funct. Ecol..

[38] Haigh A., O’Riordan R.M., Butler F. (2014). Hedgehog *Erinaceus europaeus* mortality on Irish roads. Wildl. Biol..

[39] Rautio A., Isomursu M., Valtonen A., Hirvelä-Koski V., Kunnasranta M. (2016). Mortality, diseases and diet of European hedgehogs (*Erinaceus europaeus*) in an urban environment in Finland. Mammal. Res..

[40] Wright P.G.R., Coomber F.G., Bellamy C.C., Perkins S.E., Mathews F. (2020). Predicting hedgehog mortality risks on British roads using habitat suitability modelling. PeerJ.

[41] Goransson G., Karlsson J.L. (1976). Road mortality of the hedgehog *Erinaceus europaeus* in southern Sweden. Fauna Flora.

[42] Wembridge D.E., Newman M.R., Bright P.W., Morris P.A. (2016). An estimate of the annual number of hedgehog (*Erinaceus europaeus*) road casualties in Great Britain. Mammal. Commun..

[43] Huijser M.P., Bergers P.J.M. (2000). The effect of roads and traffic on hedgehog (*Erinaceus europaeus*) populations. Biol Conserv.

[44] Holsbeek L., Rodts J., Muyldermans S. (1999). Hedgehogs and other animal traffic victims in Belgium: Results of a countrywide survey. Lutra.

[45] Becher S.A., Griffths R. (1998). Genetic differentiation among local populations of the European hedgehog (*Erinaceus europaeus*) in mosaic habitats. Mol. Ecol..

[46] Doncaster C.P., Rondinini C., Johnson P. (2001). Field test for environmental correlates of dispersal in hedgehogs *Erinaceus europaeus*. J. Anim. Ecol..

[47] Grilo C., Zanchetta Ferreira C., Revilla E. (2015). No evidence of a threshold in traffic volume affecting road-kill mortality at a large spatio-temporal scale. Environ. Impact Assess. Rev..

[48] Curto M., Winter S., Seiter A., Schmid L., Scheicher K. (2019). Application of a SSR-GBS marker system on investigation of European hedgehog species and their hybrid zone dynamics. Ecol. Evol..

[49] Rasmussen S., Nielsen J., Jones O.R., Berg T.B., Pertoldi C. (2020). Genetic structure of the European hedgehog (*Erinaceus europaeus*) in Denmark. PLoS ONE.

[50] Collinson W.J., Parker D.M., Bernard R.T.F., Reilly B.K., Davies-Mostert H.T. (2014). Wildlife road traffic accidents: A standardized protocol for counting flattened fauna. Ecol. Evol..

